# Leisure Time Habits and Levels of Physical Activity in Children and Adolescents

**DOI:** 10.3390/children11070883

**Published:** 2024-07-21

**Authors:** Juan-José Mijarra-Murillo, Beatriz Polo-Recuero, Adrián Solera-Alfonso, Alberto Arribas-Romano, Miriam García-González, Sofía Laguarta-Val, José Manuel Delfa-de-la-Morena

**Affiliations:** 1 Department of Physical Therapy, Occupational Therapy, Rehabilitation and Physical Medicine, Health Sciences Faculty, Universidad Rey Juan Carlos, 28922 Madrid, Spain; juanjose.mijarra@urjc.es (J.-J.M.-M.); beatriz.polo.recuero@urjc.es (B.P.-R.); adrian.solera@urjc.es (A.S.-A.); alberto.arribas@urjc.es (A.A.-R.); miriam.garciag@urjc.es (M.G.-G.); jose.delfa@urjc.es (J.M.D.-d.-l.-M.); 2 Cognitive Neuroscience, Pain, and Rehabilitation Research Group (NECODOR), Faculty of Health Sciences, Universidad Rey Juan Carlos, 28922 Madrid, Spain

**Keywords:** sedentary lifestyle, exercise, schoolchildren, adolescence, behavior, schoolwork, screen use, health

## Abstract

Background/Objectives: Childhood and adolescence are important stages of life for acquiring healthy habits. There is a high prevalence of sedentary lifestyles worldwide during these ages, which negatively impacts health. This is attributed, in part, to excessive time spent engaging in sedentary behaviors. The aim of this study was to assess the time spent on sedentary behaviors and their relationship with physical activity levels in children and adolescents in the Community of Madrid. Methods:A total of 26,729 participants aged 10–17 from various schools and institutes took part in this study. The International Physical Activity Questionnaire Short Form (IPAQ-SF) was used, and they were asked about the time they spent on different leisure time habits (specific sedentary behaviors and organized physical activity). A generalized linear model was used to analyze the association between the time spent in sedentary activities and the time spent in physical activity. Results: The results revealed that children and adolescents engage in low levels of physical activity and most of them spend considerable time in sedentary behaviors such as studying, watching television, or using social media. Completing school homework (Coef: 1.23, 95% CI: −0.51 to 2.97, *p* = 0.167) or using social media for more than 2 h (Coef: 1.29, 95% CI: −2.98 to 0.40, *p* = 0.133) compared to not dedicating time to them did not show a significant association with daily physical activity time. Watching television for more than 2 h was associated with a decrease of 2.60 min (95% CI: −4.41 to −0.78, *p* = 0.005). Thus, no or only irrelevant associations were found between time spent in sedentary activities and physical activity time. Conclusions: Despite the drawbacks of spending time engaging in sedentary behaviors, they seem to be compatible with physical activity levels. Therefore, it is important to continue research on physical activity adherence strategies to promote overall health and well-being.

## 1. Introduction

Physical activity is widely recognized in the scientific literature for its diverse benefits on physical and mental well-being [1,2,3]. Physical activity also contributes to the prevention of future chronic diseases and metabolic disorders [4], as well as to a reduction in mortality from these, thus also contributing to the control of public health expenditure [5].

Childhood and adolescence are critical periods for habit formation, highlighting the importance of the early establishment of physical activity habits [6]. Getting into the habit of physical activity at an early age has a positive influence on maintaining this healthy habit in adulthood [7]. However, spending too much time engaging in sedentary behaviors appears to have more negative than positive consequences in childhood and adolescence. At these ages, spending more time using screens is associated with detrimental body composition and physical fitness, as well as unfavorable social behavior and lower self-esteem [8].

The World Health Organization [9] recommends that children and adolescents aged 5–17 years should engage in a weekly average of 60 min of moderate to vigorous physical activity per day. According to WHO data, in 2016 [10], 81% of children and adolescents (between 11 and 17 years old) did not meet the minimum recommendations for being physically active. Likewise, the latest Spanish Health Survey, published by the Ministry of Health, Social Services and Equality, shows that 12% of participants between 5 and 14 years of age do not engage in any physical activity, and 45% of participants between 15 and 24 years of age do not engage in any or very little activity [11]. The decline in physical activity habits among children and adolescents is of great concern [12]. At these ages, levels of physical inactivity seem to increase progressively with age, which may be due in part to the large number of sedentary behaviors that have become established in recent times, such as the use of new technologies [13,14,15,16]. Electronic devices and social media provide convenience and entertainment, but they also pose a risk of addiction and consume significant amounts of free time [17,18]. This significant decline in physical activity levels occurs from an early age and appears to be greater in the female sex [19]. Compliance with the recommendations known as “The 24-Hour Movement Guidelines” (physical activity time, screen use, and sleep habit) has been associated with positive health effects. However, it appears that the majority of young people do not comply with this recommendation, making it necessary to insist on this and to investigate strategies to address this global health issue [20].

In this context, understanding leisure and free-time habits, and their interaction with physical activity, is important to prevent a sedentary lifestyle and promote health-oriented behavior [3,21]. In their study, Aulbach et al. [22] affirm the need to evaluate more data on the leisure behaviors of adolescents and the options they choose to spend their free time on, such as, for example, the use of screens, which occupies a relevant place [23]. The use of social media is here to stay. Therefore, it is very important to know whether this is really a bad influence on being physically active. Demonstrating that this may not be related could be relevant to focusing on other possible determinants of physical inactivity. In addition, the results of the study by Solís García and Borja González [24] show that more than half of the school children surveyed (mean age 8.92 years) spend more than 1 h a day doing homework, and more than a quarter spend more than 2 h a day doing homework. It would be relevant to test whether this time spent by students on homework is useful in determining being physically active or not.

There are many studies that deal with the level and habits of physical activity in schoolchildren, but very few deal with its relationship with the time spent on sedentary behavior. In fact, in the Community of Madrid there is only one study that focuses only on students aged 15–17 years [25], and in the city of Madrid the analysis was of the entire population, with no specific data for children and adolescents [26]. Therefore, the present study focuses on childhood and adolescence, covering all stages of education at these ages (primary school, secondary school, and college). In addition, it seems relevant to study the influence of possible factors such as educational cycle, sex, school ownership, population size, and average annual income of each municipality due to studies that reflect the need to verify the specific characteristics of school groups [27].

It seems that improving physical activity levels and reducing time spent in sedentary behaviors appears to have positive effects. In a systematic review [28] on the relationship between physical activity, sedentary behaviors, and the quality of life in children and adolescents, associations were found that show the more physical activity, the better the quality of life; additionally, the more time spent in sedentary behaviors, the lower the quality of life, but it would be interesting to see whether more time spent in sedentary behaviors determines being physically inactive. This has been another reason that gives meaning to the purpose of our study and leads us to consider the following hypothesis: children and adolescents who spend many hours doing schoolwork, watching television, or using social media are more physically inactive than those who spend fewer hours performing these types of sedentary behaviors. Secondarily, we also consider that sociodemographic variables such as age, sex, school ownership, average income of the municipality, and population size influence physical activity levels and leisure time habits.

For these reasons, our main objective is to analyze the possible association between physical activity time and sedentary activity time. As a secondary objective, we intend to test the influence of sex, age, center ownership, and the socioeconomic and demographic characteristics of the different municipalities on the time spent in physical activity and leisure time habits.

## 2. Materials and Methods

### 2.1. Study Design

This research employed a cross-sectional case study design in Madrid using a quantitative methodological approach and a variety of data collection tools. The survey was conducted individually under the supervision of the research team. Data collection was conducted between February 2019 and January 2020. The information was collected and recorded through an anonymization process to protect the identities of the respondents. Ethical approval for all procedures was granted by the Ethics Committee of the Universidad Rey Juan Carlos (registration number 1306201809818).

### 2.2. Participants

Through a non-probabilistic sampling, 26,729 students (13,491 boys and 13,238 girls) between 10 and 17 years of age from public, private, and subsidized schools in municipalities and cities of the Community of Madrid (Spain) of different sizes and different per capita incomes were evaluated.

Inclusion criteria included all students enrolled in the aforementioned participating centers who were in class at the time of the survey and who were willing to participate. Exclusion criteria included students whose survey responses did not meet the completion rules.

### 2.3. Instruments and Variables

The International Physical Activity Questionnaire (IPAQ-SF) developed by Craig et al. [29] was used to assess physical activity levels. This questionnaire has previously been used in studies with children and adolescents [30,31,32,33], and its use has been validated in the Spanish population [34]. Questionnaires are widely used measurement instruments when a sample is very large. The IPAQ is a validated questionnaire commonly used to assess physical activity levels. Participants reported moderate to vigorous activity in the past 7 days, which can be calculated as meeting the minimum physical activity recommendations established by the WHO. To be considered physically active, participants had to reflect that they performed on average at least 60 min of moderate to vigorous physical activity per day. Anything less than that number was considered a non-physically active participant.

In addition, participants were asked about the time they spent on sedentary behaviors, such as doing schoolwork, watching TV, and using social media, as well as the time they spent on organized physical activity. Television viewing and social media use are sedentary behaviors associated with screen time but are distinguished by the passive nature of television viewing compared to the interactive and participatory aspects of social media use. It is worth clarifying that time spent on sedentary activities (homework, watching TV, and using social media) and organized physical activity was collected through a question on how much time they spent on these activities. To facilitate the response, the following options were given categorically: none, less than 1 h, between 1 and 2 h, and more than 2 h.

These categorical variables were classified according to the total sample (*N* = 26,729) and according to five characterization variables with their respective categories: educational cycle (5th and 6th year of primary school: from 10 to 12 years old; 1st and 2nd year of secondary school: from 12 to 14 years old; 3rd and 4th years of secondary school: from 14 to 16 years old; and college: from 16 to 17 years old), sex (male and female), ownership of the school (public and private/subsidized), municipal average income (low average, high average, high) and population (rural: less than 5000 inhabitants; urban: 5000 to 99,999 inhabitants; metropolitan: 100,000 or more residents). We have defined this classification according to the data from the National Institute of Statistics (INE).

### 2.4. Data Analysis

The statistical analyses were conducted using STATA (IC 16.1, StataCorp LLC, Lakeway Drive, College Station, TX, USA). Descriptive statistics and frequencies were employed to report daily physical activity time, prevalence of physical inactivity, and time spent in sedentary and organized activities.

ANOVA and Student’s *t*-tests were used to estimate differences in physical activity time by grade, sex, ownership, income, and population size when residuals were normally distributed, and variances were homogeneous. Kruskal–Wallis and Wilcoxon tests were used when these assumptions were violated. Normality was assessed using the Shapiro–Wilk test and Q-Q plots, while homogeneity of variances was evaluated with Levene’s test. The effect size of the differences was reported using eta^2^ (η^2^), Cohen’s d, and the rank correlation coefficient statistic. The association between time spent in sedentary activity and organized physical activity, collected as categorical variables, with sociodemographic variables and physical inactivity was analyzed by the chi-square test, and Cramér’s V was used to calculate the effect size. The strength of association was categorized as negligible, small, medium, or large based on the degrees of freedom, as outlined in the Appendix A (Table A1).

Associations between daily physical activity time and time spent in different sedentary activities were explored with separate generalized linear models (GLMs). Physical activity time was entered as the dependent variable, sedentary activity time as the independent variable, and possible confounding factors of course, sex, ownership, income, and population size were controlled for. A *p*-value of less than 0.05 was considered significant.

## 3. Results

### 3.1. Levels of Physical Activity

A total of 66.61% of participants did not meet the WHO minimum physical activity standards. The percentage of physically active participants, and the average number of minutes of physical activity, was lower as the school year increased, among females, in public schools, and in low-income cities or cities with larger populations. However, the effect size of the differences in these variables was very low except for sex, which was moderate (Table 1).

**Figure A1:**
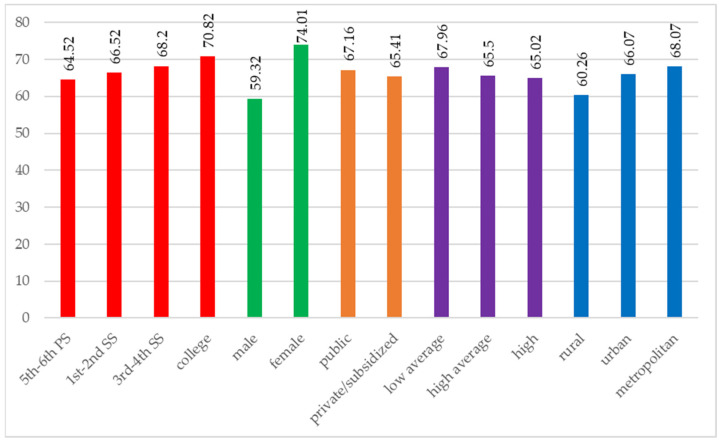
Percentages of physically inactive subjects according to each variable (PS, primary school; SS, secondary school).

Figure A1 (Appendix B) illustrates the data pertaining to the percentage of physically inactive subjects based on each characterization variable.

### 3.2. Sedentary Behaviors

#### 3.2.1. Schoolwork

The results show that the proportion of time spent on homework increases as the educational cycle progresses among women, private/subsidized schools, and municipalities with higher incomes and populations greater than 100,000 (Appendix A, Table A2). All associations were statistically significant despite small effect sizes, except for the training cycle, which had a medium effect size. The results indicate that time spent studying has a statistical association with daily physical activity time. Compared to those who do not spend time studying, it was found that spending between 0 and 1 h studying is associated with an increase of 4.48 min in daily physical activity (95% CI: 2.56 to 6.40, *p* < 0.001). Similarly, spending between 1 and 2 h in the study is associated with a 2.57 min increase in daily physical activity (95% CI 0.91 to 4.22, *p* = 0.002). However, these increases, although statistically significant, are too small to be considered practically relevant. Spending more than 2 h in the study did not show a statistically significant association with daily physical activity (Coef: 1.23, 95% CI: −0.51 to 2.97, *p* = 0.167) (Table 2).

**Figure 1:**
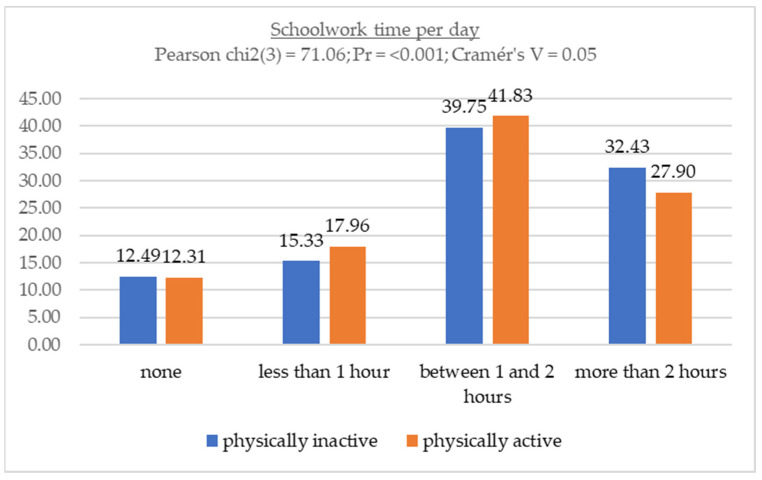
Association between schoolwork time and physical activity levels.

Figure 1 shows the relationship between homework time and physical activity level. Considering the relationship between these sedentary behaviors and IPAQ-SF responses according to WHO recommendations, the results show that the proportion of time spent doing homework is very similar between physically active and physically inactive individuals.

#### 3.2.2. Television

The results for TV viewing time show a gradual increase with age, especially for men, public institutions, high-income, and populated municipalities (Appendix A, Table A3). All associations were statistically significant, the effect size for training cycle was quite medium, and the rest were small or negligible.

The results indicate that time spent watching television has statistically significant but practically insignificant associations with daily physical activity time. Compared to those who do not spend time watching television (reference category), it was found that spending between 0 and 1 h watching television was associated with a 2.61 min increase in daily physical activity (95% CI 0.77 to 4.45, *p* = 0.006). Spending between 1 and 2 h watching TV did not show a statistically significant association with daily physical activity (Coef: 0.13, 95% CI: −1.66 to 1.93, *p* = 0.886). Spending more than 2 h watching television was associated with a 2.60 min decrease in daily physical activity (95% CI: −4.41 to −0.78, *p* = 0.005). Although these differences are statistically significant, the changes in minutes of physical activity are small (Table 3).

**Figure 2:**
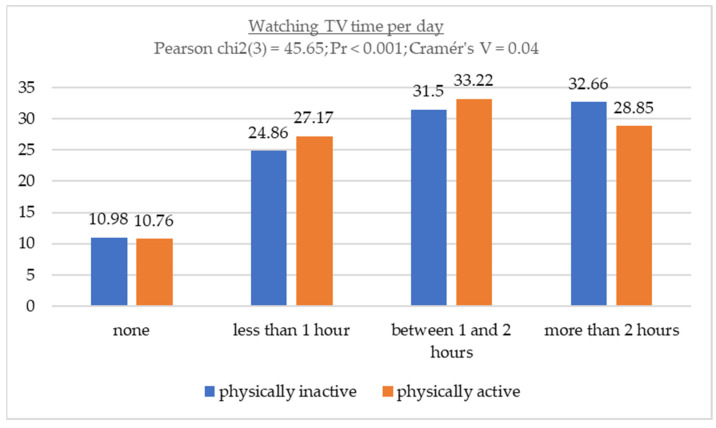
Association between watching TV time and physical activity levels.

Figure 2 shows the relationship between TV viewing time and physical activity level. Furthermore, when examining the relationship between these sedentary behaviors and IPAQ-SF responses according to WHO recommendations, it became clear that the proportion of time spent in each category was very similar between physically active and physically inactive individuals.

#### 3.2.3. Social Media

The findings regarding time spent using social media indicate that the percentages increase with advancing educational cycles, among females, in public institutions, and in municipalities with low average income levels and high population densities (Appendix A, Table A4). These associations were statistically significant, with most exhibiting a small effect size. Notably, a large effect size was observed for the educational cycle.

The results indicate that time spent using social media shows some association but of little practical relevance to daily physical activity time. Compared to those who do not spend time using social media, it was found that spending between 0 and 1 h on social media is associated with a 2.47 min increase in daily physical activity (95% CI: 0.76 to 4.17, *p* = 0.005). Spending between 1 and 2 h (Coef: 1.42, 95% CI: −0.29 to 3.14, *p* = 0.104) and more than 2 h (95% CI: −2.98 to 0.40, *p* = 0.133) on social media did not show a statistically significant association with daily physical activity. Although doing between 0 and 1 h of social media is associated with more physical activity time compared to doing nothing, the difference in minutes is small and has little practical relevance (Table 4).

**Figure 3:**
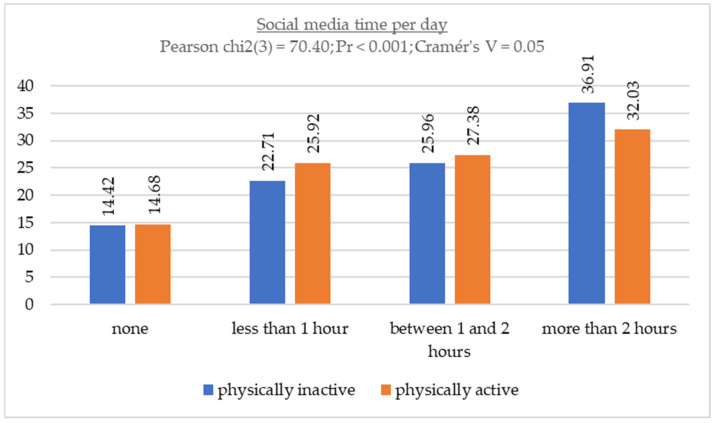
Association between social media time and physical activity levels.

Figure 3 illustrates the relationship between time spent using social media and physical activity levels. Furthermore, considering the relationship between these sedentary behaviors and responses to the IPAQ-SF in accordance with WHO recommendations, the results suggest that there is minimal difference in the percentages of time spent between physically active and physically inactive individuals.

### 3.3. Organized Physical Activity Habits

In examining responses related to formal physical sports activity habits, it is noteworthy that 28.56% of students are not enrolled in any formal physical activity program. With respect to age, there is a progressive increase in the percentage of participants not enrolled in any activity of this nature as educational cycles advance. Additionally, higher percentages of individuals not enrolled in organized physical activity were observed among females, in public institutions, and in municipalities with lower income levels and larger populations (Appendix A, Table A5). These associations were statistically significant, although effect sizes were generally small, except in relation to educational level, where it was medium.

## 4. Discussion

### 4.1. Levels of Physical Activity

Our findings raise urgent concerns about the physical activity levels of children and adolescents in the Community of Madrid. In our study, responses to the IPAQ-SF questionnaire showed that 66.61% of the participants fail to meet WHO’s physical activity recommendations. Comparable figures from studies conducted in other countries corroborate this trend [35,36]. Our data, when compared to the national context, present a more positive outlook. Guthold et al. [37] and Tremblay et al. [38] reported higher levels of physical inactivity for Spanish children and adolescents as a whole. This suggests that the Community of Madrid achieves higher levels of physical activity among its youth population compared to the overall data for the rest of the national territory, as indicated in the latest SIVFRENT-J report analyzing health habits [25]. In this regard, a report by Díaz-Ollalla [26] entitled Estudio de Salud de la Ciudad de Madrid 2018 shows that the proportion of sedentary leisure time in Madrid (30%) is significantly lower than nationally (37.8%). Although these data are insufficient, they seem to support the idea that residents of Madrid have more physically active habits than the general Spanish population, reflecting their generally less sedentary lifestyle.

Our study also shows higher levels of physical inactivity as age and the educational cycle progress. In this regard, our results coincide with those of the Consejo Superior de Deportes [39], where it was observed that there is a progressive increase in physical inactivity with age, or with the powerful study in Spanish territory by Gómez et al. [40], who presented a physical inactivity of 55.5% in childhood and 69.9% in adolescence. Studies have also been found in which this worrying increase in the levels of physical inactivity coincides between the end of the primary school stage and the beginning of the secondary school stage, such as that of Franco et al. [41]. It seems that the greater responsibility and autonomy that adolescents are beginning to be given in the management of their free time may have an influence [42], and it may also be due to the fact that children engage in physical activity just for the sake of playing, while adolescents tend to engage in physical-sports activity mainly if there is a competitive factor, which may reduce the time spent in physical activity [43].

Furthermore, with regard to differences by sex, a significantly higher percentage of physical inactivity is observed in girls (74.01%) than in boys (59.32%), something that coincides with international studies [10,27,44] and studies in the Community of Madrid itself, such as that of Laguna-Nieto et al. [45], which also presented much higher data on physical inactivity for women. It appears that one of the reasons may be due to a perception of inequality with respect to men and a fear of being judged [46].

All these data are available from Jaramillo-Guzmán and Ávila-Mediavilla [47], and Ordoñez et al. [48] and Polo-Recuero et al. [49] confirm the importance of the various strategies and interventions proposed which complete the theme of physical education. These strategies aim to address low levels of physical activity in children and adolescents, with a particular focus on females and middle and high school levels.

### 4.2. Sedentary Behaviors

Physical activity depends not only on the hours of sedentary life, but also on multiple factors (e.g., previous physical activity, community sports, parent support, support from others) that we can influence and perhaps are neglecting by focusing too much on the problem of screen abuse [50].

Our results show that the characterization variable most associated with the performance of sedentary behaviors (homework, watching television, and using social media) is the educational cycle, as with the greater the educational cycle, the greater the time spent on these three behaviors.

In the current study, we observed a progressive increase according to age in the number of subjects who spend more than 2 h per day on homework, which practically doubles from primary school to high school (23.39% vs. 50.26%), which seems to reflect that a greater amount of this type of activity is carried out as the educational cycle progresses. There is research showing the impact of time spent on homework reducing formal and free time for physical activity [40], since, among other factors, more hours are dedicated to homework as the grade increases [51]. However, our study reflects that this behavior does not seem to determine being physically active by itself. In addition, there are recent studies that show how physical activity at these ages could benefit academic performance, so it seems advisable to combine them [52,53,54,55].

Regarding daily television viewing time, several studies reflect an increase in screen use with age [56,57] and longer times in boys than in girls [27,58], something that coincides with our results. However, regarding the relationship between daily screen viewing time and being physically active, although there are different studies and interventions in which the time children spend in front of screens is reduced in favor of their physical activity [59,60,61] and reduction in their sedentary time [62], in our results it has been seen that this behavior does not determine physical inactivity. A systematic review of 4559 papers showed some variables to be consistently associated with children’s physical activity, such as engaging in physical activity, an absence of elimination games, and scheduling physical activity in daily programming [63].

In terms of the time spent on the use of social media by children and teenagers, we can see the progressive increase in use, especially when they move to high school, which is something that may be related to the fact that, according to different studies, the average age of possession or access to mobile phones in Spain is between 11 and 12 years old [64,65], which coincides with the end of the primary school stage. Furthermore, it seems that those adolescents who rely heavily on social media spend more time in front of screens in their leisure time and do not foster their socioemotional skills, as in Gaspar et al. [66]. Likewise, social media seems to favor a worrying addiction and influence the increase in cyberbullying [67]. Regardless of these negative aspects, despite social media, the time spent in the use of social media has not been associated with physical activity.

The association of sedentary behaviors with the rest of the characterization variables (school ownership, average income, and size of the municipality) is negligible. And, as mentioned in the three sedentary behaviors, when analyzing the relationship between the time spent in these behaviors and the levels of physical activity, our results show that there is little association. This finding implies that the general view of the matter changes, on the contrary of what was thought, to the idea that sedentary behavior displaces light activity throughout adolescence [68]; the present findings call into question whether these sedentary behaviors determine the fate of not meeting the minimum WHO recommendations regarding being physically active or not. This seems to highlight the importance of not attributing the blame for the high level of physical inactivity at these ages to these types of behaviors and to paying more attention to promoting physical activity, as they are perfectly compatible with each other.

### 4.3. Organized Physical Activity Habits

Finally, taking into account the analysis of organized physical activity, the present study confirms that there is less participation in out-of-school physical activities as students get older. One of the main reasons why students choose a physical sports activity seems to be due to social influence, especially from parents and family members in general [69], which seems to justify why adolescents sign up less, since they are more independent. Furthermore, Portela-Pino et al. [70] identified laziness and fatigue as the main barriers in the adolescent stage. Another reason is the family and work conciliation; parents often find it necessary to enroll their children in extracurricular activities during the primary school stage in order to cover the family work schedule [71].

According to gender, it is observed that boys do more formal physical activity than girls, as already detailed in other studies [72], and this may be due to the fact that it is girls who encounter greater barriers to participating in these activities [70].

In addition, it is worth highlighting the influence of being enrolled in organized physical activity and being physically active. As explained by Salmensalo et al. [73], the preference for sport or organized and routine physical activity is inversely related to physical inactivity. In fact, another study [61] found that young people who participate in grassroots sport are more likely to continue to engage in physical activity and sport in the future and establish healthy active leisure habits. This seems to be the key to maintaining healthy physical activity habits over time. For all these reasons, the main finding is the little relationship between being physically active and carrying out sedentary behaviors. These two concepts have been misleadingly and wrongly associated in our society, and just a few studies, such as Biddle et al. [74], have refuted and clarified the issue. This idea highlights the need to encourage physical activity programs and promote participation in them from an early age, as they are crucial in the acquisition of habits, in order to encourage adherence to physical activity in later stages.

### 4.4. Practical and Academic Implications

Although it is important to stress the importance of regulating the time spent in sedentary activities, seeking the balance recommended by the 24 h movement guidelines, our study invites us to focus not so much on this as on motivating children and adolescents to engage in physical activity. Therefore, it is necessary to establish strategies such as, for example, a greater supply of physical activity from municipalities and the government, such as bio-healthy parks and accessible programs to promote physical activity and sports among citizens.

All in all, how do we sell physical activity to adolescents? Our study may inspire more motivational messages about the benefits not in terms of judgment of the fact that they spend too much time in front of screens, but with the positive message that spending time on social media, television, (something inherent to their time in life, which is more difficult to influence as adults who grew up in different societies)… is not incompatible with the practice of physical activity.

### 4.5. Limits

Our study is not without limitations:

1. Despite presenting such a large sample with different sociodemographic characteristics, the sample may not be representative of the Community of Madrid, since probability sampling was not carried out.
2. Longitudinal studies will certainly be necessary in order to know the evolution over time, since the determinants of physical inactivity related to sedentary behaviors are quite complex. We do not know how these habits may evolve in subsequent periods of education, such as university. We are aware of the results of studies in these populations, on 595 university students [75], in which neither physical activity nor sedentary behavior demonstrated a significant relation with the level of life satisfaction. But we consider the subject to be controversial, and it is essential to continue to focus research on it, since this same study found that sedentary behavior at weekends was negatively related to objective and subjective quality of life as well as to dimensions including intimacy, safety, and the communicative aspect of the quality of life.
3. Although a questionnaire validated in the scientific literature (IPAQ-SF) was used, due to the large sample size, it was not feasible to use more objective methods of data collection, such as accelerometry.
4. Another limitation could be to have categorized the responses on leisure time habits, instead of collecting it as a continuous variable, although recall bias may also influence this type of data collection.
5. In spite of the fact that the data collection was not conducted in special educational centers, no information was obtained as to whether there were any participants who might hold grounds for exclusion from the study due to physical limitations.

### 4.6. Strenghts

Among the strengths of this study are the large significant sample of participants and the standardization of the measurement of the variables through questionnaires. These questionnaires were administered under the supervision of the research team, who were available to the participants to resolve any doubts that arose at the time of completion.

### 4.7. Future Perspectives

Future lines of research could also focus on motivations. Deci and Ryan’s self-determination theory [76] can be considered as a starting point. This theory classifies motivation into several types (intrinsic motivation, extrinsic motivation, or demotivation), emphasizing the relevant influence of the role of motivation in the adherence to the performance of any activity, in this case, in the performance of physical sport activity. It is very useful for understanding human behavior, promoting intrinsic motivation, satisfying psychosocial needs, and helping to improve physical and mental well-being.

Thus, finding out what types of physical sports activities children and adolescents engage in, both free and organized, as well as their relationship with motivation towards them could be another point that could help to clarify certain aspects in order to carry out the necessary strategies for adherence to this type of healthy activity.

## 5. Conclusions

Despite the manifold benefits associated with an active lifestyle, sedentary habits such as homework, television viewing, and social media usage are prevalent among children and young people in the Community of Madrid, particularly as they progress through the educational cycle. However, our study did not find a strong association between levels of physical activity and these sedentary behaviors, indicating their compatibility. It appears that individuals can engage in both physical activity and sedentary behaviors simultaneously.

Finally, there is a need to explore strategies that promote adherence to physical activity, both free and organized, to meet physical activity recommendations and promote overall health and well-being.

## Figures and Tables

**Table 1:** Physical activity levels.

		*N* (%)	Physically Active %	Physically Inactive %	Daily Physical Activity (min) Median (IQR)	Differences between Groups		
						Statistic	*p* Value	Effect Size
General		26,729 (100)	33.39	66.61	42.86 (21.43, 68.57)			
Course	5th-6th PS	11,122 (41.61)	35.44	64.56	42.86 (25.71, 72.86)	Chi^2^ = 247.26	<0.001	η^2^ = 0.01
	1st-2nd SS	6306 (23.59)	33.48	66.52	42.86 (18.57, 68.57)			
	3rd-4th SS	6073 (22.72)	31.8	68.2	38.57 (17.14, 68.57)			
	college	3228 (12.08)	29.18	70.82	34.29 (17.14, 64.21)			
Sex	male	13,455 (50.43)	40.685	59.32	50.71 (25.71, 77.14)	Z = 31.87	<0.001	r = 0.20
	female	13,223 (49.57)	25.993	74.01	34.29 (42.86, 24.29)			
Ownership	public	18,206 (68.11)	32.84	67.16	41.43 (20.68.57)	Z = −4.12	<0.001	r = 0.03
	private/subsidized	8523 (31.89)	34.59	65.41	42.86 (21.43, 69.29)			
Income	low average	12,776 (47.80)	32.04	67.96	40 (18.57, 68.57)	Chi^2^ = 49.34	<0.001	η^2^ = 0.00
	high average	10,214 (38.21)	34.5	65.5	42.86 (21.43, 70)			
	high	3739 (13.99)	34.98	65.02	42.86 (24.29, 72.86)			
Population size	rural	1628 (6.09)	39.74	60.26	47.14 (25.71, 77,14)	Chi^2^ = 42.15	<0.001	η^2^ = 0.00
	urban	13,190 (49.35)	33.93	66.07	42.86 (21.43, 68.57)			
	metropolitan	11,911 (44.56)	31.93	68.07	40.71 (20, 68.57)			

Min, minutes; IQRSD, interquartile range standard deviation; PS, primary school; SS, secondary school.

**Table 2:** Results of the generalized linear model for estimating the association between physical activity time and schoolwork time.

Variable	Category	Coefficient	Standard Error	Z	*p*-Value	95% Confidence Interval
Schoolwork time (reference: Nothing)						
	0–1 h	4.48	0.98	4.58	<0.001	2.56 to 6.40
	1–2 h	2.57	0.84	3.04	0.002	0.91 to 4.22
	More than 2 h	1.23	0.89	1.38	0.167	−0.51 to 2.97
Course (reference: 5th and 6th PS)						
	1st and 2nd SS	−3.53	0.68	−5.16	<0.001	−4.88 to −2.19
	3rd and 4th SS	−5.95	0.69	−8.59	<0.001	−7.31 to −4.59
	College	−9.39	0.87	−10.77	<0.001	−11.10 to −7.68
Sex (reference: Male)						
	Female	−14.90	0.52	−28.49	<0.001	−15.92 to −13.87
Ownership (reference: Public)						
	Private	3.21	0.57	5.64	<0.001	2.09 to 4.33
Income (reference: Lower class)						
	High average	2.91	0.60	4.84	<0.001	1.73 to 4.09
	High	5.43	0.84	6.44	<0.001	3.78 to 7.08
Population Size (reference: Rural)						
	Urban	−7.71	1.19	−6.47	<0.001	−10.05 to −5.38
	Metropolitan	−8.27	1.17	−7.09	<0.001	−10.56 to −5.98
_cons		64.26	1.27	50.77	<0.001	61.77 to 66.74

PS, primary school; SS, secondary school.

**Table 3:** Results of the generalized linear model for estimating the association between physical activity time and TV time.

Variable	Category	Coefficient	Standard Error	Z	*p*-Value	95% Confidence Interval
TV time (reference: Nothing)						
	0–1 h	2.61	0.94	2.78	0.006	0.77 to 4.45
	1–2 h	0.13	0.92	0.14	0.886	−1.66 to 1.93
	More than 2 h	−2.60	0.92	−2.81	0.005	−4.41 to −0.78
Course (reference: 5th and 6th PS)						
	1st and 2nd SS	−3.26	0.69	−4.75	<0.001	−4.60 to −1.91
	3rd and 4th SS	−5.44	0.70	−7.82	<0.001	−6.81 to −4.08
	College	−9.25	0.86	−10.72	<0.001	−10.94 to −7.56
Sex (reference: Male)						
	Female	−15.47	0.52	−29.66	<0.001	−16.49 to −14.45
Ownership (reference: Public)						
	Private	2.98	0.57	5.23	<0.001	1.87 to 4.10
Income (reference: Lower class)						
	High average	2.91	0.60	4.84	<0.001	1.73 to 4.09
	High	5.51	0.84	6.54	<0.001	3.86 to 7.16
Population Size (reference: Rural)						
	Reference: Rural					
	Urban	−7.50	1.19	−6.28	<0.001	−9.84 to −5.16
	Metropolitan	−7.96	1.17	−6.82	<0.001	−10.24 to −5.67
_cons		66.43	1.30	51.07	<0.001	63.89 to 68.98

PS, primary school; SS, secondary school.

**Table 4:** Results of the generalized linear model for estimating the association between physical activity time and social media time.

Variable	Category	Coefficient	Standard Error	Z	*p*-Value	95% Confidence Interval
Social media time (reference: Nothing)						
	0–1 h	2.47	0.87	2.84	0.005	0.76 to 4.17
	1–2 h	1.42	0.88	1.63	0.104	−0.29 to 3.14
	More than 2 h	−1.29	0.86	−1.50	0.133	−2.98 to 0.40
Course (reference: 5th and 6th PS)						
	1st and 2nd SS	−3.29	0.70	−4.70	<0.001	−4.66 to −1.92
	3rd and 4th SS	−5.33	0.73	−7.31	<0.001	−6.75 to −3.90
	College	−8.84	0.90	−9.86	<0.001	−10.60 to −7.09
Sex (reference: Male)						
	Female	−15.01	0.52	−28.98	<0.001	−16.03 to −13.99
Ownership (reference: Public)						
	Private	3.02	0.57	5.30	<0.001	1.91 to 4.14
Income (reference: Lower class)						
	High average	2.83	0.60	4.70	<0.001	1.65 to 4.01
	High	5.31	0.84	6.31	<0.001	3.66 to 6.96
Population Size (reference: Rural)						
	Urban	−7.54	1.19	−6.32	<0.001	−9.88 to −5.20
	Metropolitan	−8.14	1.17	−6.98	<0.001	−10.42 to −5.85
_cons		65.65	1.23	53.24	<0.001	63.24 to 68.07

PS, primary school; SS, secondary school.

## Data Availability

The data presented in this study are available on request from the corresponding author. Data are unavailable due to privacy or ethical restrictions.

## Appendix A

**Table A1:** Cramer’s V effect size interpretation [77].

Degrees of Freedom	Negligible	Small	Medium	Large
1	Less than 0.10	0.10 < 0.30	0.30 < 0.50	0.50 or more
2	Less than 0.07	0.07 < 0.21	0.21 < 0.35	0.35 or more
3	Less than 0.06	0.06 < 0.17	0.17 < 0.29	0.29 or more
4	Less than 0.05	0.05 < 0.15	0.15 < 0.25	0.25 or more
5	Less than 0.04	0.05 < 0.13	0.13 < 0.22	0.22 or more

**Table A2:** Schoolwork time per day.

		None (%)	Less than 1 h (%)	Between 1 and 2 h (%)	More than 2 h (%)	Pearson’s Chi-Square	*p*-Value	Cramer’s v
General	(*N* = 26,675)	12.43	16.21	40.44	30.92			
Course	5th-6th PS (*n* = 11,101)	15.29	18.74	42.59	23.39	1.0 × 10^3^	<0.001	0.11
	1st-2nd SS (*n* = 6283)	12.57	14.66	42.62	30.14			
	3rd-4th SS (*n* = 6064)	10.55	16.00	38.24	35.21			
	college (*n* = 3227)	5.86	10.94	32.94	50.26			
Sex	male (*n* = 13,418)	14.32	18.90	41.46	25.32	489.19	<0.001	0.14
	female (*n* = 13,207)	10.38	13.47	39.46	36.69			
Ownership	public (*n* = 18,158)	13.28	16.78	40.15	29.79	69.28	<0.001	0.05
	private/subsidized (*n* = 8517)	10.61	14.99	41.07	33.32			
Income	low average (*n* = 12,764)	12.25	16.82	40.18	30.75	97.59	<0.001	0.04
	high average (*n* = 10,172)	13.19	16.33	41.63	28.84			
	high (*n* = 3739)	10.97	13.80	38.11	37.12			
Population size	rural (*n* = 1627)	15.80	18.81	42.04	23.36	104.56	<0.001	0.04
	urban (*n* = 13,146)	13.49	15.31	39.69	31.52			
	metropolitan (*n* = 11,902)	10.80	16.85	41.06	31.28			

PS, primary school; SS, secondary school.

**Table A3:** Watching TV time per day.

		None (%)	Less than 1 h (%)	Between 1 and 2 h (%)	More than 2 h (%)	Pearson’s Chi-Square	*p*-Value	Cramer’s v
General	(*N* = 26,680)	10.90	25.63	32.08	31.39			
Course	5th-6th PS (*n* = 11,100)	14.99	29.67	30.52	24.82	782.13	<0.001	0.10
	1st-2nd SS (*n* = 6286)	9.91	24.64	31.99	33.46			
	3rd-4th SS (*n* = 6071)	6.95	20.56	22.44	39.05			
	college (*n* = 3223)	6.21	23.24	35.03	35.53			
Sex	male (*n* = 13,431)	9.53	21.61	33.45	35.42	370.57	<0.001	0.12
	female (*n* = 13,199)	12.20	29.76	30.72	27.32			
Ownership	public (*n* = 18,166)	11.81	24.78	31.61	31.80	65.91	<0.001	0.05
	private/subsidized (*n* = 8514)	8.97	27.46	33.06	30.50			
Income	low average (*n* = 12,762)	10.59	25.74	32.18	31.48	91.56	<0.001	0.04
	high average (*n* = 10,179)	11.46	25.97	33.46	29.11			
	high (*n* = 3739)	10.43	24.36	27.95	37.26			
Population size	rural (*n* = 1627)	15.24	31.84	30.42	22.50	135.22	<0.001	0.05
	urban (*n* = 13,150)	11.63	25.13	31.20	32.04			
	metropolitan (*n* = 11,903)	9.51	25.34	33.27	31.88			

PS, Primary School; SS, Secondary School.

**Table A4:** Social media time per day.

		None (%)	Less than 1 h (%)	Between 1 and 2 h (%)	More than 2 h (%)	Pearson’s Chi-Square	*p*-Value	Cramer’s v
General	(*N* = 26,679)	14.51	23.78	26.43	35.28			
Course	5th-6th PS (*n* = 11,104)	25.57	35.02	22.26	20.15	4.0 × 10^3^	<0.001	0.22
	1st-2nd SS (*n* = 6280)	9.82	24.09	29.36	36.72			
	3rd-4th SS (*n* = 6070)	5.04	14.71	29.14	51.10			
	college (*n* = 3225)	3.35	11.88	29.98	54.79			
Sex	male (*n* = 13,424)	14.93	23.54	27.41	34.12	24.99	<0.001	0.03
	female (*n* = 13,205)	13.99	24.05	25.47	36.49			
Ownership	public (*n* = 18,162)	15.54	23.04	25.83	35.59	64.95	<0.001	0.05
	private/subsidized (*n* = 8517)	12.29	25.37	27.72	34.61			
Income	low average (*n* = 12,767)	13.65	23.64	26.41	36.30	39.23	<0.001	0.03
	high average (*n* = 10,173)	15.38	23.96	27.27	33.39			
	high (*n* = 3739)	15.03	23.80	24.23	36.94			
Population size	rural (*n* = 1627)	21.08	32.33	23.36	23.23	262.26	<0.001	0.07
	urban (*n* = 13,146)	15.70	21.92	25.79	36.60			
	metropolitan (*n* = 11,906)	12.29	24.68	27.57	35.47			

PS, Primary School; SS, Secondary School.

**Table A5:** Organized physical activity time per day.

		None (%)	Less than 1 h (%)	Between 1 and 2 h (%)	More than 2 h (%)	Pearson’s Chi-Square	*p*-Value	Cramer’s v
General	(*N* = 26,729)	28.56	56.98	12.15	2.31			
Course	5th-6th PS (*n* = 11,122)	23.42	64.43	10.16	1.99	514.27	<0.001	0.08
	1st-2nd SS (*n* = 6306)	29.91	55.26	12.67	2.16			
	3rd-4th SS (*n* = 6073)	32.69	50.65	13.60	3.06			
	college (*n* = 3228)	35.87	46.53	15.27	2.32			
Sex	male (*n* = 13,455)	23.97	58.77	14.49	2.78	362.39	<0.001	0.12
	female (*n* = 13,223)	33.23	55.15	9.79	1.83			
Ownership	public (*n* = 18,206)	29.50	56.44	11.79	2.26	27.17	<0.001	0.03
	private/subsidized (*n* = 8523)	26.55	58.11	12.92	2.42			
Income	low average (*n* = 12,776)	31.39	54.41	11.99	2.22	141.18	<0.001	0.05
	high average (*n* = 10,214)	26.90	59.37	11.66	2.07			
	high (*n* = 3739)	23.43	59.21	14.04	3.32			
Population size	rural (*n* = 1628)	28.69	59.71	9.64	1.97	65.22	<0.001	0.03
	urban (*n* = 13,190)	26.83	58.78	12.26	2.13			
	metropolitan (*n* = 11,911)	30.46	54.60	12.38	2.56			

PS, primary school; SS, secondary school.
